# Inappropriate shock in a subcutaneous cardiac defibrillator due to residual air

**DOI:** 10.1002/ccr3.1009

**Published:** 2017-06-06

**Authors:** Shawn Lee, Nektarios Souvaliotis, Davendra Mehta, Ranjit Suri

**Affiliations:** ^1^ Mount Sinai St. Luke's Hospital New York City New York

**Keywords:** Defibrillator, ICD, inappropriate shock, subcutaneous air, subcutaneous ICD

## Abstract

Inappropriate shock due to residual air in subcutaneous implantable cardiac defibrillators is not a well‐known complication. Obtaining overpenetrated X‐rays, recognizing electrocardiogram findings, limiting blunt finger dissection, and switching to sense at another vector are techniques which might lead to avoidance of unnecessary wound exploration or device removal.

## Case Presentation

A 55 year‐old man with hypertrophic cardiomyopathy and nonsustained ventricular tachycardia underwent a subcutaneous cardiac defibrillator (S‐ICD; Cameron Health/Boston Scientific) implant for primary prevention of sudden cardiac death. A S‐ICD was chosen, as he was relatively young, had no indications for pacing, and pre‐ECG screening showed that he was appropriate for this device.

**Figure 1:**
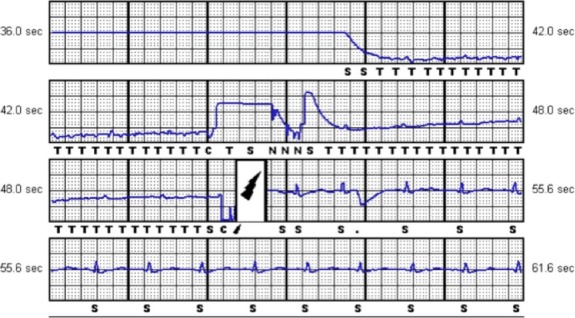
Electrocardiograms from S‐ICD interrogation: discernable but blunted QRS complexes interpreted as ‘tachy’ are seen prior to shock.

**Figure 2:**
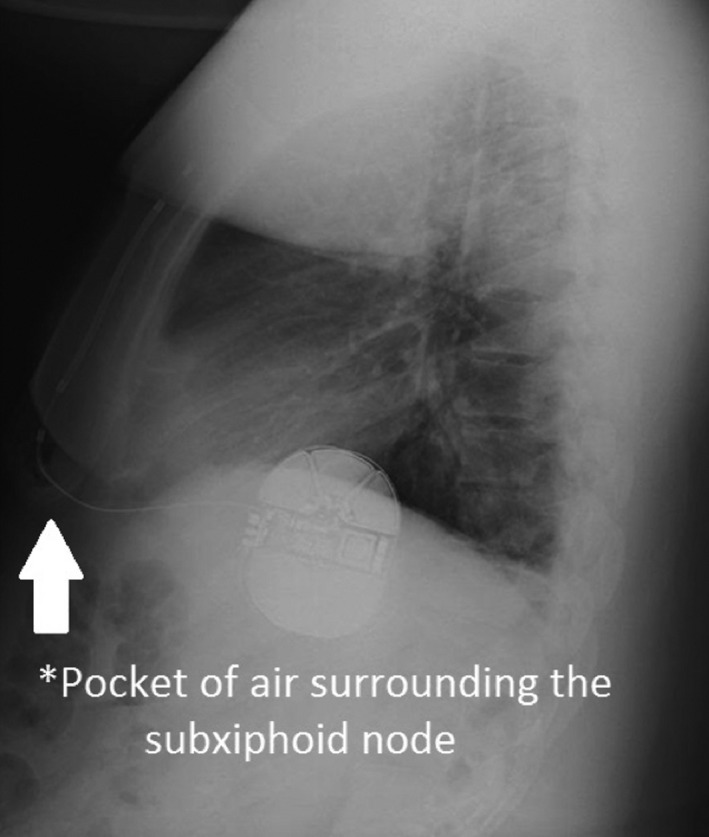
Lateral chest film showing air surrounding subxiphoid node.

**Figure 3:**
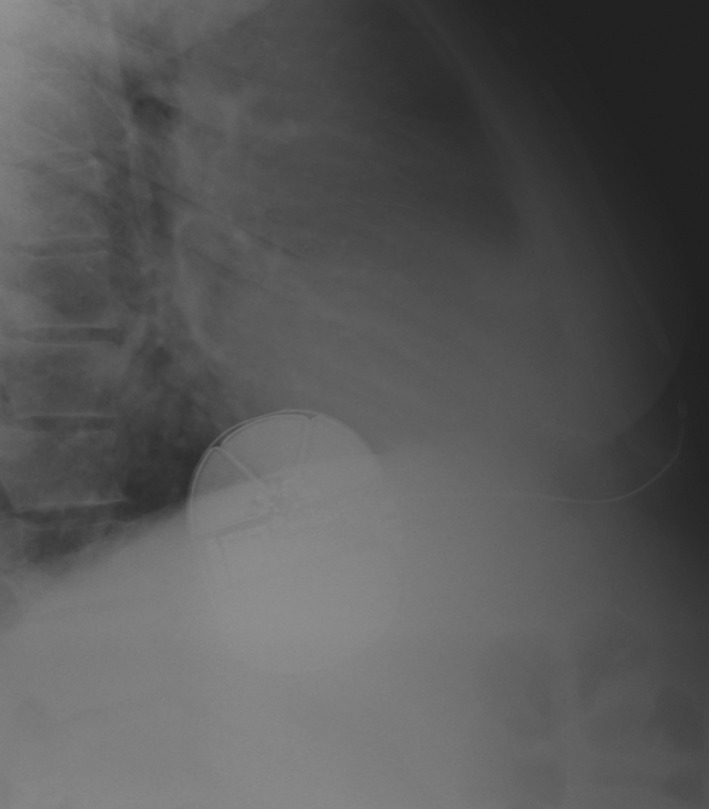
Lateral chest film 2 weeks post‐op showing resolution of air around subxiphoid node.

A standard three‐incision technique was performed. After the coils and generators were placed and closed in three layers, ventricular fibrillation was induced and detected in the primary vector. Sinus rhythm was effectively restored with a submaximal 65‐J shock. A post‐op PA chest X‐ray confirmed device and lead placement. Roughly 6 h after the procedure on the telemetry floor, the patient received a shock. Device interrogation revealed oversensing in the primary vector (Fig. 1). Handgrip maneuver and manipulation (manual percussion) over the leads of the device did not reproduce any noise. Moreover, the impedance of the device was within normal limits, suggesting no lead fracture. An overpenetrated PA and lateral chest X‐ray showed air around the subxiphoid node (Fig. 2). The device was reprogrammed to sense at the secondary vector, and the patient was discharged. He followed up in electrophysiology clinic 2 weeks later with no complaints of shock, and a repeat lateral chest film showed resolution of air around the subxiphoid node (Fig. 3).

## Discussion

As the Food and Drug Administration approved the use of the S‐ICD in 2012 1, its usage and popularity have grown. The EFFORTLESS S‐ICD Registry (an international database of S‐ICD users) has demonstrated that S‐ICDs have appropriate performance as well as similar outcomes in quality of life and similar rates of inappropriate shock (roughly 7% in a mean follow‐up of 1 year) compared to transvenous ICDs 2, 3. Follow‐up of these patients after a mean of 2 years demonstrated that programming of sensing with the primary vector reduced the risk of inappropriate shock. Most inappropriate shocks were due to oversensing of T waves; other causes included oversensing of “low amplitude signals,” supraventricular tachycardias and noncardiac oversensing (which included electromagnetic interference) 4, 5. For example, Frommeyer et al. 6 reported an inappropriate shock from a S‐ICD due to artifact from electrical interference from a street lantern.

There has been sparse literature about oversensing from artifact due to subcutaneous air. In 1979, entrapped air within the device pocket was first reported as a cause for complete cessation of myocardial stimulation in a unipolar pacemaker 7. Since then, air has been recognized as a potential insulator that prevents direct tissue contact with the electrode. In fact, Boston Scientific recently updated its user's manual for S‐ICDs and recommends that residual air should be evacuated prior to closing and suturing to optimize sensing and deliver therapy. Flushing the sternal track with saline, massaging the skin over the leads, and proper suturing over the sensing electrodes have been suggested as techniques to reduce dead space air within subcutaneous tissue 8.

Zipse et al. 9 reported the first case of inappropriate shock in a S‐ICD due to subcutaneous air surrounding the distal electrode, causing oversensing in the secondary vector. Another case by Yap et al. 10 reported subcutaneous air surrounding the proximal electrode, causing oversensing in the alternate vector. In both cases, shocks were delivered within 48 hours of device implantation. The devices were reprogrammed to sense at a different vector, which was effective at preventing any further inappropriate shocks. Resolution of air around the electrodes was confirmed on lateral chest films after several days in both cases.

S‐ICDs are traditionally implanted by a three‐incision technique, but an alternative approach employing only two incisions (the superior parasternal incision is avoided) has also been used. Does an extra incision with the traditional technique risk air introduction? Review of literature is conflicting. A prospective cohort by Knops et al. 11 evaluated the safety and efficacy of the two‐incision technique in S‐ICD implantations. After an 18‐month follow‐up, 39 patients with implantation using a two‐incision technique had no inappropriate sensing 11. However, Chinitz et al. 8 reported two cases of inappropriate shock due to subcutaneous air surrounding electrodes in patients whom the two‐incision technique was utilized and argue that the three‐incision technique reduces this complication 8. Finally, despite their own experience of an inappropriate shock after a two‐incision technique, Gamble et al 12. argue that the two‐incision technique is less likely to result in air around the distal electrode as they had found radiographic evidence of subcutaneous air surrounding the proximal electrode only.

In our discussions with Boston Scientific, we discussed this patient and the overall issue and collaboratively have the following recommendations:

1. It is important to recognize the unique electrocardiogram in this case, which is the characteristic of artifact secondary to subcutaneous air. Air causes a rise in impedance leading to a baseline shift from the isoelectric point (seen in Fig. 1 in the 48.0–55.6 sec bracket) and decreased amplitude of QRS signaling leading to oversensing. Recognizing these features on interrogation would avoid unnecessary wound exploration after an inappropriate shock.
2. Surgical technique is important, and it is imperative to use the tunneling tool (provided in Boston Scientific's kit) to tunnel the coils through, and limit blunt finger dissection. The tunneling tool is much thinner than the operator's finger, and the smaller diameter of the tool reduces the chance of air coming through. Flushing saline through the sheath and massaging air along the tract would help to expel any air out prior to closing.
3. Finally, we recommend overpenetrated PA and lateral chest films immediately after S‐ICD placement to detect the presence of air. If air is detected, the device should be reprogrammed to sense at another vector until air resorbs, usually without intervention, in 1–2 weeks.

## Conflict of Interest

None declared.

## Authorship

SL: was responsible for writing up initial drafts, obtaining patient consent, and coordinating correspondences and meetings between authors. Nektarios Souvaliotis: was responsible for obtaining electrocardiogram tracings and images and assistance in writing of initial drafts and subsequent edits. DM: was responsible for the literature search and assistance in subsequent edits and finalizing manuscript. RS: was responsible for the literature search; and assistance in subsequent edits and finalizing manuscript.
